# An intelligent sport shoe to prevent ankle inversion sprain injury

**DOI:** 10.1186/1757-1146-5-S1-K6

**Published:** 2012-04-10

**Authors:** Daniel TP Fong

**Affiliations:** 1 Department of Orthopaedics and Traumatology, Faculty of Medicine, The Chinese University of Hong Kong, Hong Kong

## Background

Ankle sprain injury is common in sports. This study presents an intelligent sport shoe to prevent it.

## Materials and methods

(1) Sensing: Five subjects performed various sporting motions with data collected from a plantar pressure system to reconstruct the ankle supination torque determined from a motion capture system with a force plate. Validation test on another five subjects was conducted. (2) Identification: Six subjects performed simulated sub-injury and non-injury trials with the dorsal foot kinematics measured by 8 wearable motion sensors. Data was used to train a support vector machine to establish a mathematics algorithm for identification, which was validated on another 6 subjects, with an expected accuracy of 90%. An uni-axial gyrometer was placed at the position with the best accuracy for identifying ankle sprain hazard, with a threshold suggested from a database of ankle inversion velocity from real injury incidents, sub-injury trials and non-injury motions. (3) Correction: Myoelectric stimulations with different delay time (0, 5, 10 and 15ms) were delivered to the peroneal muscles of 10 subjects performing unanticipated sub-injury trials in a laboratory. The effect was quantified by the heel tilting angle and its velocity as determined by a motion analysis system.

## Results

(1) Sensing: a system with 3 pressure sensors was developed to monitor the ankle supination torque with overall root mean square error as 6.91Nm, which was 6% of the peak values recorded (Fong et al, 2008a). (2) Identification: A method with one gyrometer at the heel to identify hazardous motion with 91.3% accuracy was developed (Chu et al, 2010). (3) Correction: significant reduction of the heel tilting angle and velocity from 18 to 9-13 degrees and from 200-250 to 140-170 deg/s was achieved.

## Conclusions

An intelligent anti-sprain sport shoe with a 3-step intelligent system is successfully invented, and is soon being ready for commercialization.
